# Dr. Laurence Turnbull

**Published:** 1900-12

**Authors:** 


Dr. Laurence Turnbull, of Philadelphia, died in that city October 24, 1900, aged 79 years. He was noted as an otologist and was the first in this country to trephine the mastoid for ear disease.

